# Qualification of a quantitative stability indicating potency assay for mRNA-LNP vaccine candidates

**DOI:** 10.12688/gatesopenres.16382.1

**Published:** 2026-05-14

**Authors:** ANAN BZAMI, Marcus Estrada, Tim Schofield, Jessica A. White

**Affiliations:** 1 PATH, Seattle, WA, 8103,, USA; 2 CMC Science, Germantown,, MD, 20876, USA

**Keywords:** Potency, mRNA-LNP, ELISA

## Abstract

Potency assays assess the functional integrity of a target antigen to ensure that the final drug product is consistent between the commercial and clinical trial lots. Existing mRNA-LNP vaccine potency assays require complex quantification methods, such as flow cytometry, which are not widely adaptable to routine use by low- and middle-income country (LMIC) manufacturers or National Regulatory Agency (NRA) laboratories. To address the need for more accessible mRNA-LNP vaccine potency methods, an enzyme-linked immunosorbent assay (ELISA) was developed to quantify the target proteins produced by an mRNA-LNP vaccine after cell transfection. SARS-CoV-2 mRNA-LNP targeting the wild-type (Wuhan) strain was used as a model vaccine for the development and qualification of an ELISA method. The adaptability of the ELISA to other mRNA-LNP vaccines was demonstrated by altering the method used for quantification of a commercial Omicron mRNA-LNP vaccine. Alternative mRNA-LNP potency methods, such as western blotting, fluorescence focus assay (FFA), and direct ELISA were used as analogous methods for comparison. The developed quantitative stability-indicating ELISA is a globally accessible potency method that utilizes existing equipment and knowledge. Testing conducted on wild type SARS CoV-2 and Omicron vaccines demonstrates the adaptability of the method to other mRNA vaccine targets.

## Introduction

mRNA vaccine use was first successfully described in animals in 1990; however, application of this technology has been extended to a wide variety of targets, including cancer, HIV, influenza, rabies, Zika, and respiratory syncytial virus. ^1^^,^^2^ The SARS-CoV-2 pandemic has rapidly accelerated the development of mRNA-LNP vaccines and highlighted the potential for broad application of this platform to novel vaccine development. mRNA-LNP vaccines represent a promising approach to novel vaccine development because this technology allows for rapid, low-cost manufacturing of a vaccine candidate with significant safety. ^1^

Potency, used for both product release and stability monitoring, is a critical attribute of every vaccine and is required for the regulatory approval and release of all products. Several factors may affect mRNA-LNP vaccine potency and have been identified as critical quality attributes with corresponding complex analytical methods developed for monitoring, including mRNA sequence integrity, poly A tail length, 5’ capped percentage, mRNA-LNP size, dsRNA content, etc. ^1^^,^^3^^–^^6^ However, characterization of these critical attributes does not confirm transcription and appropriate folding of the target protein in the host cell, which is required to elicit a protective immune response. Potency assays for mRNA-LNP vaccines need to satisfactorily assess the functional integrity of the target protein to ensure that it achieves and retains immunocompetence. ^3^^,^^4^^,^^7^^,^^8^ A potency assay that is able to quantify the target protein and indicates stability may obviate the need to routinely monitor all the critical quality attributes of an mRNA-LNP vaccine candidate.

mRNA-LNP potency methods require protein expression in cell culture after transfection of mRNA with LNP. After transfection, any generated protein is measured using either qualitative methods such as western blotting or quantitative complex methods such as flow cytometry or fluorescence focus assay (FFA). ^9^^–^^13^ Both western blot and flow cytometry methods present challenges when used as routine potency methods, especially in low- and middle-income country (LMIC) manufacturing settings. For example, flow cytometry methods are challenging to standardize for validation and require costly technical equipment. In addition to the assay challenges described above, there are no standardized approaches to mRNA-LNP transfection. Variations in the cell lines used, age of the cells, and time after transfection all contributed to variability in the amount of protein generated and, ultimately, the final potency measured. Owing to the multiple steps required for conducting *in vitro* mRNA-LNP potency assays, the generation of consistent and globally comparable results that meet the validation requirements using common standards is critical to mRNA-LNP vaccine development.

This study describes the development and qualification of an adaptable sandwich enzyme-linked immunosorbent assay (ELISA) method for the quantification of target proteins expressed *in vitro* using a model mRNA-LNP vaccine. Through the project, key assay parameters were evaluated, and target specifications for critical assay steps were identified to optimize the method. A SARS-CoV-2 vaccine targeting the wild-type spike variant was used as a model vaccine for method development. The adaptability of the potency method was demonstrated by applying it to a commercial mRNA-LNP vaccine against the Omicron variant (Moderna, XBB1.5). Alternate potency methods, including western blotting, direct ELISA, and fluorescence focus assay (FFA), were tested in parallel with the sandwich ELISA. This study describes the successful qualification of a quantitative sandwich ELISA, which is stable and can be adapted for use with other mRNA-LNP vaccine targets.

## Materials and methods

### mRNA, mRNA-LNP and antibody reagents

mRNA and mRNA-LNP targeting the wild-type (NCBI NC_045512.2) and Omicron BA.4/5 (NCBI OR529199) variants were purchased from Genescript USA for use in method development. A commercial Moderna mRNA-LNP vaccine targeting Omicron XBB.1.5 (2023-2024) was purchased for use in method development.

Multiple antibodies targeting specific SARS CoV-2 variants were purchased and screened for use in method development (R&D Systems, Sino Biological, MHRA/NIBSC). Antibodies were selected based on their sensitivity to the wild-type and Omicron variants and global accessibility. Antibody sensitivity was determined using ELISA. Purified recombinant proteins, purchased from Sino Biological, from wild-type, Omicron BA 4/5, and Omicron XBB variants were coated onto an ELISA plate. The antibodies were tested across dilutions of the recombinant protein.

### Cell culture

HEK293 cells (BEI Resources) and HEPG2 cells (ATCC) were cultured according to ATCC culture guidelines for each cell line. Cells were thawed in DMEM (HEK293; Thermo Fisher Scientific) or EMEM (HEP2G; Gibco) containing 20% fetal bovine serum (FBS; Gibco) but were maintained in 10% FBS with Penicillin/Streptomycin/Glutamine (Thermo Fisher Scientific) in a tissue culture incubator set to 37°C with 5% CO _2_ with 95-100% humidity.

### mRNA-LNP transfection and extraction

HEK293 or HEPG2 cells were grown to approximately 90-100% confluency in 75 cm ^2^ flasks prior to plating on cell culture plates. Three flasks were combined, counted, and resuspended in 10 mL of fresh 10% FBS DMEM or EMEM media. Cells were diluted to 5 × 10 ^5^ or 1 × 10 ^6^ cells per well to coat three cell culture plates, 12-well or 24-well plates, respectively, with 1 mL of cells per well with or without APOE3 (Acro Biosystems) at 0, 1, 2, or 4 μg/mL.

After plating, cell culture plates were transfected 18–24 h later with either wild-type mRNA-LNP or Omicron BA4/5 mRNA-LNP. Each mRNA-LNP was diluted in sterile phosphate buffered saline (PBS) (KD Medical) prior to adding 0.1 mL to the appropriate wells and gently shaken by hand before being placed in the tissue culture incubator. mRNA with Rmesfect transfection reagent (OZ Biosciences) was also tested. Cell culture plates were incubated overnight (22–24 h).

Expressed proteins were extracted by carefully aspirating media out of each well and adding 0.1 mL of extraction buffer made of 1% Triton-X 100 (Sigma-Aldrich) in PBS (KD Medical). RIPA buffer (Thermo Fisher Scientific) and cell extraction buffer (Invitrogen) were also tested. Plates were shaken at 500 RPM for 30 min, collected into individual 1.5 mL tubes, and frozen at -80°C prior to testing by sandwich ELISA and western blotting.

### FFA and direct ELISA

Cell lines (HEK293 or HEPG2) were diluted to the appropriate cell plating concentration (6 × 10 ^4^, 4 × 10 ^4^ or 2.67 × 10 ^4^ cells per well in PBS and 0.15 mL) and added to sterile 96-well black walled, clear bottom, tissue culture treated plates (Corning). HEK293 cells were plated with either 2 μg/mL poly-D-lysine (PDL; Sigma-Aldrich) or 1.5 μg/mL polyethylenimine (PEI; Sigma-Aldrich) to increase cell attachment to the plate. Cells were washed, fixed with 4% formaldehyde (Thermo Fisher Scientific), permeabilized with 0.1% Triton X-100, then diluted primary antibody was added and shaken overnight at room temperature (RT). Plates were washed, and then fluorescently labeled secondary antibody diluted in PBS was added.

Plates were washed with PBS the next day using a plate washer (Biotek) with a flow rate set to 3, the slowest setting for dispensing and aspiration. cells were then fixed using 0.1 mL of 4% formaldehyde and set to incubate for 15 min at RT. Waste was manually aspirated and placed into hazardous waste. Plates were washed with plate washer and then permeabilized with 0.1 mL of 5% BSA (Thermo Fisher Scientific) + 0.1% Triton-X 100 in PBS and set to incubate for 1 h at RT. primary antibody was diluted to 15 μg/mL in 5% BSA with 0.1% Triton-X 100 and incubated overnight at RT shaking at 500 RPM. After incubating overnight, the plates were washed with PBST using a plate washer at a flow rate of 3. Anti-mouse IgG 647 antibody (Thermo Fisher Scientific) was diluted to 2 μg/mL in 1% BSA in PBS and 0.1 mL was added to each well. The plates were shaken for 1 min at 500 RMP and then transferred to a humidified 5% CO _2_ incubator at 37°C for 1 h. The plates were washed and 0.1 mL) was added to each well prior to reading the number of fluorescent cells for FFA on the Spectramax i3X plate reader with the Minimax attachment (Molecular Devices) at 713 nm. Labeled cells were detected using Softmax Pro version 7.1.2 (Molecular Devices) at one site per well.

For the direct ELISA, the same conditions as described for the FFA method were applied, but the amount of protein was measured by reading the total fluorescent signal present in each well of the assay plate using an ELISA plate reader with a fluorescence detector (Molecular Devices Spectramax i3X).

The sensitivity of FFA and direct ELISA to changes in mRNA-LNP concentration or stability were evaluated by testing mRNA-LNP samples subjected to heat stress at 75°C for 10 min. All tests were conducted in triplicate.

### Sandwich ELISA

We began by establishing the sandwich ELISA method using wildtype SARS-CoV-2 (2019-nCoV) Spike S1 recombinant protein (Acro biosystems) then extended to subsequent protein extract screening. Half-area 96-well high binding (Corning) were coated with diluted anti-spike antibody 40150-D006 (Sino Biological) in 1x PBS to a final concentration of 1 μg/mL. The plates were sealed and incubated overnight (16-24 hours) at 2–8°C.

After incubation, plates were washed with 0.2 mL/well of 1X PBST (KD medical) using a plate washer and then blocked with 0.1 mL/well of 1% BSA (Thermo Fisher Scientific) in 1X PBS assay buffer for 1 h at RT. The recombinant protein was diluted 10-fold in assay buffer and serially diluted 2-fold across the ELISA plate for a total of 7 dilutions. The cell extracts were removed at -80°C and centrifuged at 12,000 × g for 10 min. The supernatant was collected and diluted 10-fold (wild-type) or 20-fold (Omicron BA.4/5) in assay buffer and serially diluted 1.67-fold across the ELISA plate for a total of 11 dilutions. 0.05 mL/well of test sample dilutions were added to the plate in duplicate. After 1 hour at RT the plate is washed and 0.05 mL/well of primary antibody 101119-B (175 MHRA/NIBSC mAb) is added at a final concentration of 1 μg/mL then 0.05 mL/well of a 1:4000 dilution of secondary antibody (anti-mouse HRP mAb; Millipore). RT tetramethylbenzidine (TMB; Sigma-Aldrich) 0.05 mL/well was added to the plate prior to shaking at 500 rpm for 15–30 seconds and placing in the dark to incubation for 15 ± 1 minutes at RT. 0.05 mL/well of 1N H _2_SO _4_ (JT baker) was added to stop the reaction. The ODs were measured at a wavelength of 450 nm within five minutes of addition of the stopping reagent using a Spectramax i3X plate reader. The statistical software STATLIA MATRIX was used to calculate relative potency. Acceptance criteria included a blank well OD <0.2, 15% CV replicates, and system suitability.

The sensitivity of the Sandwich ELISA to mRNA-LNP concentration or stability was evaluated by testing mRNA-LNP samples subjected to heat stress at 75°C for 10 min. All tests were conducted in triplicate.

### Western blot

Western blot analysis was performed by testing a 2-fold dilution range starting at 0.5 μg/well mRNA-LNP as the standard and a high and low load of mRNA-LNP for the test samples (0.25 μg/well, 0.5, and 1 μg/well). Samples were prepared in a total volume of 50 μL by adding the appropriate amounts of 4X loading dye (Invitrogen), water for injection (WFI; cytiva), and test sample or standard to obtain the desired antigen load. The protein ladder was prepared by diluting 5 μL of Bio-Rad Precision plus protein all blue (Thermo Fisher Scientific) with 25 μL of 4X loading dye and 70 μL of WFI. After samples and standards cooled, 20 μL was loaded onto a premade 12-well Nupage 4-12% Bis-Tris gel (Invitrogen) with 5 μL of diluted ladder into the appropriate wells. The gel and 1X running buffer (Invitrogen) were placed into the gel apparatus and set to run at 200 V for 30 min. Proteins were then transferred onto a Nupage 0.45 um nitrocellulose transfer membrane (Invitrogen) soaked in 1X transfer buffer (Invitrogen) with 10% methanol (Sigma-Aldrich) at 30 V for 60 min. The blot was removed, rinsed with deionized water, and then placed in a tray at 150 RPM with 1X fluorescence blocking solution (Thermo Fisher Scientific) for 20 min at RT. The appropriate primary antibody (R&D system 105407) was diluted (0.05 μg/mL directly into the blocking solution, placed on an orbital shaker set to 150 RPM to incubated overnight at RT. Blots were washed three times the next day using 1X PBST wash buffer (KD Medical) and incubated for 5 min with shaking at 150 RPM on an orbital shaker. The appropriate secondary antibody (Thermo Fisher Scientific Goat Antirabbit IgG Alexa Fluor 647) was diluted 1:2000 into fresh 1X fluorescence blocking solution and shaken at 150 RPM to incubate for 1 h while being covered in foil. After incubation, the blot was washed three times with deionized water, and a wet image was acquired using a G:Box Mini 9 (Syngene) imager at 647 nm. Values were obtained using densitometry measurements using GeneTools analysis software (Syngene).

The sensitivity of western blotting to changes in mRNA-LNP concentration or stability was evaluated by testing mRNA-LNP samples subjected to heat stress at 75°C for 10 min. All tests were conducted in triplicate.

### Sandwich ELISA qualification

After finalization of the parameters for the sandwich ELISA method, qualification was conducted by two analysts conducting triplicate testing for a total of three experiments. Each analyst transfected a total of three 12-well cell culture plates in triplicate with four mRNA-LNP samples: a 0.5 μg/well standard and three test samples 0.25 μg/well, 0.5 μg/well and 1 μg/well mRNA-LNP. Each cell culture plate generated 9 ELISA plates for each assay run per operator. This process was repeated thrice for a total of 27 ELISA plates.

Qualification testing was conducted using frozen, single-use HEK293 cell aliquots. Cells were thawed for 2 min in a 37°C water bath and cultured in 20% FBS DMEM media in a 25 cm ^2^ flask (Corning). The next day, the cells were split into six 75 cm ^2^ flasks (Corning): 3 flasks contained 14 mL of media and 1 mL of cells, and three flasks contained 14.5 mL of media and 0.5 mL of cells. All six flasks were grown for three days in a tissue culture incubator set to 37°C with 5% CO _2._ Prior to selecting, flasks were plated and tested using the sandwich ELISA methods described above.

### Statistical analysis

The assay qualification study data were transformed using the natural log (ln) and analyzed per USP General Chapter <1033> *Validation of Biological Assays* using JMP ^®^ software. ^14^ Validation attribute estimates were obtained using a linear mixed effects model (MEM), where the validation sample level was treated as a fixed effect, and operator, cell culture preparation within operator, and replicate ELISA series were random effects. Estimates of relative accuracy (relative bias), repeatability, and intermediate precision were obtained at both individual and across levels. Linearity was obtained from separate modeling of ln measured potency versus ln expected potency to obtain the slope and coefficient of determination of the linear fit. Relative accuracy was evaluated using the results obtained from two operators who prepared three cell cultures each (i.e., six runs). The results are reported as percent relative bias (%RB). Repeatability (within-run variability) measures the variability of the relative potency (RP) measurements obtained within a single run of the bioassay (i.e., from a single cell culture preparation and ELISA plate dilution series), while intermediate precision (between-run variability) is the variability between runs. The results were reported as the percentage geometric coefficient of variation (%GCV).

Variance component estimates were also used to predict the variability of a reportable result for various replication strategies comprising i-operators (O), j-days (D), k-cell culture preparations per operator and day [C (OD)], and l-replicates (ELISA plates or R).

### Comparisons to a direct binding ELISA and FFA

The qualification results were combined with data obtained from direct ELISA and FFA to compare responses across levels in the assays. Direct ELISA and FFA data were comprised of three potency measurements at each of two levels, 50% and 200%, and using wild type SARS CoV-2 and Omicron mRNA-LNPs. The triplicate measurements (ln) were averaged, as were the results of triplicate plates in the qualification. An analysis of covariance (ANCOVA) was performed to obtain the linear relationships of ln measured (RP) versus ln expected RP, modeling the categorical variable method (Direct ELISA, FFA, and Qualification) and the regression slope nested in the method.

## Results

### Antibody selection

**Figure 1.**
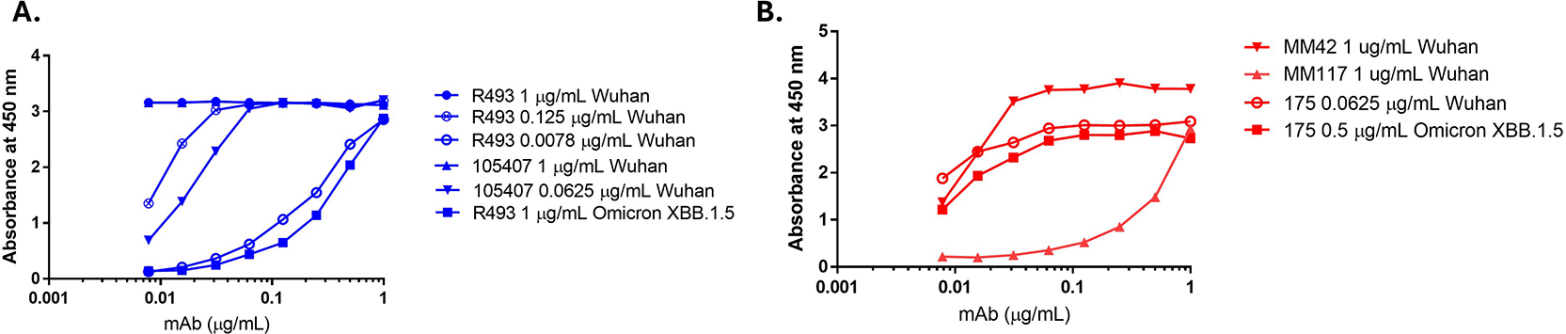
Antibody screening data by ELISA. SARS CoV-2 purified S trimer recombinant proteins (SARS-CoV-2 WT and Omicron XBB1.5; Sino Biologicals) were coated on an ELISA plate at 1, 0.125, 0.0625, and 0.0078 μg/mL for the SARS-CoV-2 WT variant or 1 and 0.5 μg/mL for the Omicron XBB 1.5 variant. Rabbit (40592-R493, Sino Biologicals) and mouse (Ms 175, MHRA) antibodies against the SARS CoV-2 S antigen were tested across all coating concentrations in 2-fold dilutions starting at 1 μg/mL.

Antibodies were selected based on their sensitivity to the Spike (S) antigen recombinant proteins from different SARS CoV-2 variants by ELISA. Antibodies were diluted across a concentration range and tested against dilutions of recombinant proteins coated on an ELISA plate. Data showing the sensitivity of two rabbit S-antigen-specific anti-spike antibodies (40592-R493 (Sino Biological) and anti-spike 105407 (R&D system)) and three mouse specific (175 (MHRA/NIBSC), MM42 (Sino Biological) and MM117 (Sino Biological)) are shown in Figure 1. Only one lot was tested for each antibody as they were monoclonals and should be limited if any lot-to-lot variation. Table 1 outlines the calculated EC50 values for each antibody. Antigen specific antibodies were selected for method development based on binding to antigen bound to ELISA plates and confirmed through sandwich ELISA, direct ELISA, FFA and western blot use.

**Table 1.** EC50 values for antibody screening.

mAb (ug/mL)	EC50
Rb493 1 ug/mL Wuhan	0.58
Rb493 0.125 ug/mL Wuhan	0.012
Rb493 0.0078 ug/mL Wuhan	0.325
Rb493 1 ug/mL Omicron XBB.1.5	0.607
Ms175 0.0625 ug/mL Wuhan	0.001
Ms175 0.5 ug/mL Omicron XBB.1.5	0.009
Rb105407 1 ug/mL Wuhan	0.58
Rb105407 0.0625 ug/mL Wuhan	0.022

### Method development

In the development of FFA, WB, direct, and sandwich ELISAs, several method parameters were evaluated, as shown in Table 2. The method development was an iterative process, and not all parameters were evaluated for each method. The details of the development of each method are described below.

**Table 2.** Assay parameters tested during method development.

Assay parameter	Variables evaluated
Cell type	HEK293 and HEPG2
Protein extraction buffers	• 1% Triton X 100 in PBS • Radioimmunoprecipitation assay buffer (RIPA buffer) (Thermo Fisher Scientific) • Cell Extraction Buffer (Thermo Fisher Scientific catalog # FNN0011) • Additional freeze thaw cycles (30 mins) • Addition of Protease inhibitor
Media additives	• ApoE3 (1,2,4 μg) • 2 μg/mL poly-D-lysine • 1.5 μg/mL polyethylenimine
Cell plating concentrations	• 96-well plates: 6X10 ^4^, 4X10 ^4^ and 2.67X10 ^4^ cells/well • 12- and 24-well plates: 1X10 ^6^ and 5x10 ^5^ cells/well
Overnight incubation after addition of mRNA-LNP	18h and 24h

### mRNA-LNP protein expression and extraction

**Figure 2.**
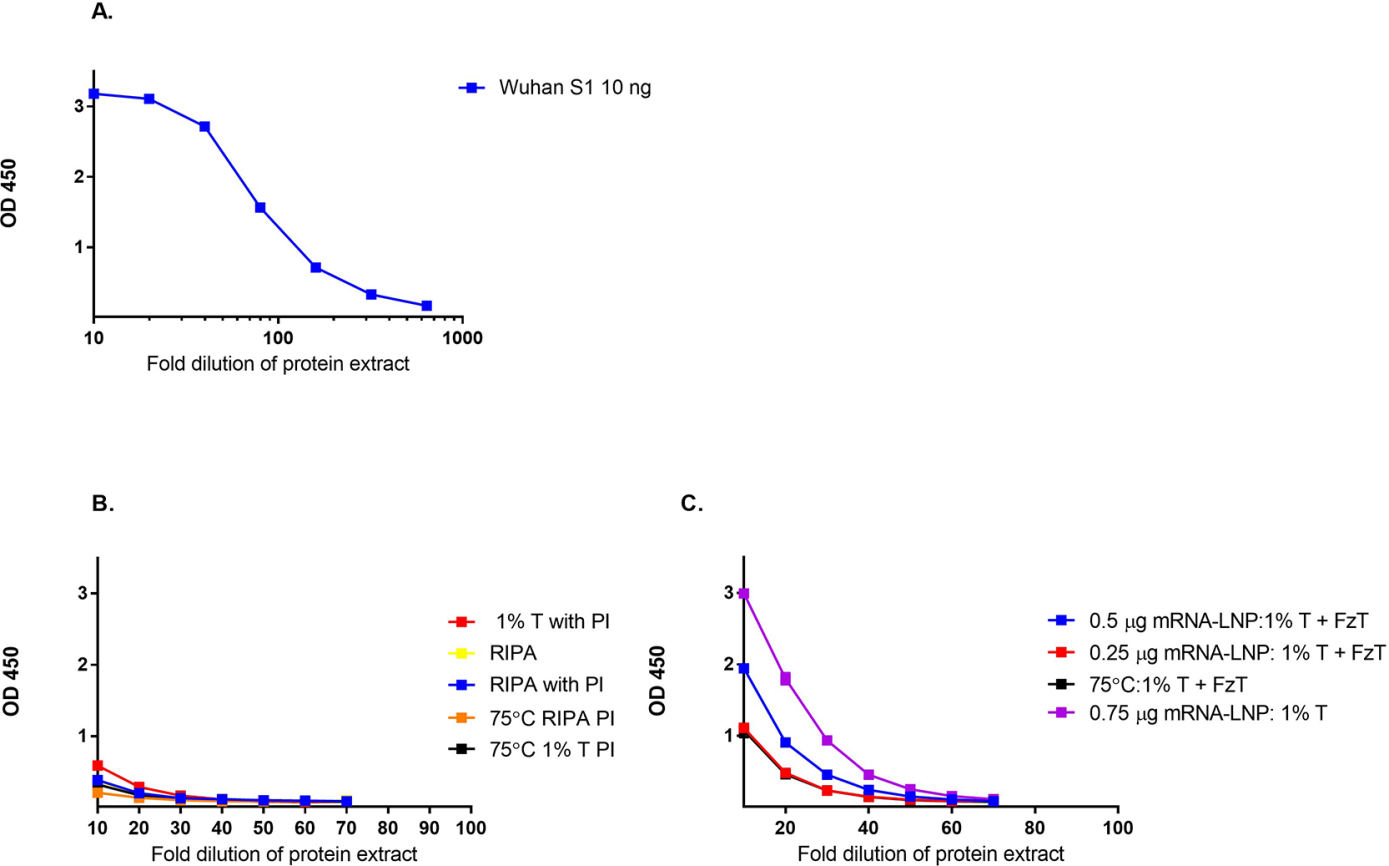
Sandwich assay parameters and the effect of cell line and extraction buffer on protein concentration. The amount of protein recovered after overnight transfection with mRNA-LNP into A) HEPG2 cells and B) HEK293 cells using different extraction buffers (1% Triton X (T); RIPA, protease inhibitor (PI), freeze thaw cycle (FzT)) was measured by Sandwich ELISA.

The sandwich ELISA used to assess protein expression was first evaluated using wildtype SARS-CoV-2 (2019-nCoV) Spike S1 recombinant protein ( Figure 2A). Proteins were expressed in both HEK-293 and HEPG2 cell lines to generate the S antigen for measurement. The amount of protein antigen generated was evaluated using sandwich ELISA. As shown in Figure 2B and 2C, HEK 293 cells generated an increased concentration of protein compared to HEPG2 cells after overnight incubation, as determined by sandwich ELISA. Different protein extraction buffers and the addition of protease inhibitors ( Figure 2) were also evaluated for their impact on the amount of protein recovered after the transfection of HEPG2 and HEK293 cells. The use of different extraction buffers or the addition of protease inhibitors after extraction did not significantly increase the amount of protein recovered from HEPG2 cells compared to HEK293 cells. Based on these results, HEK293 cells were selected for the further development of this method.

### FFA and direct ELISA

**Figure 3.**
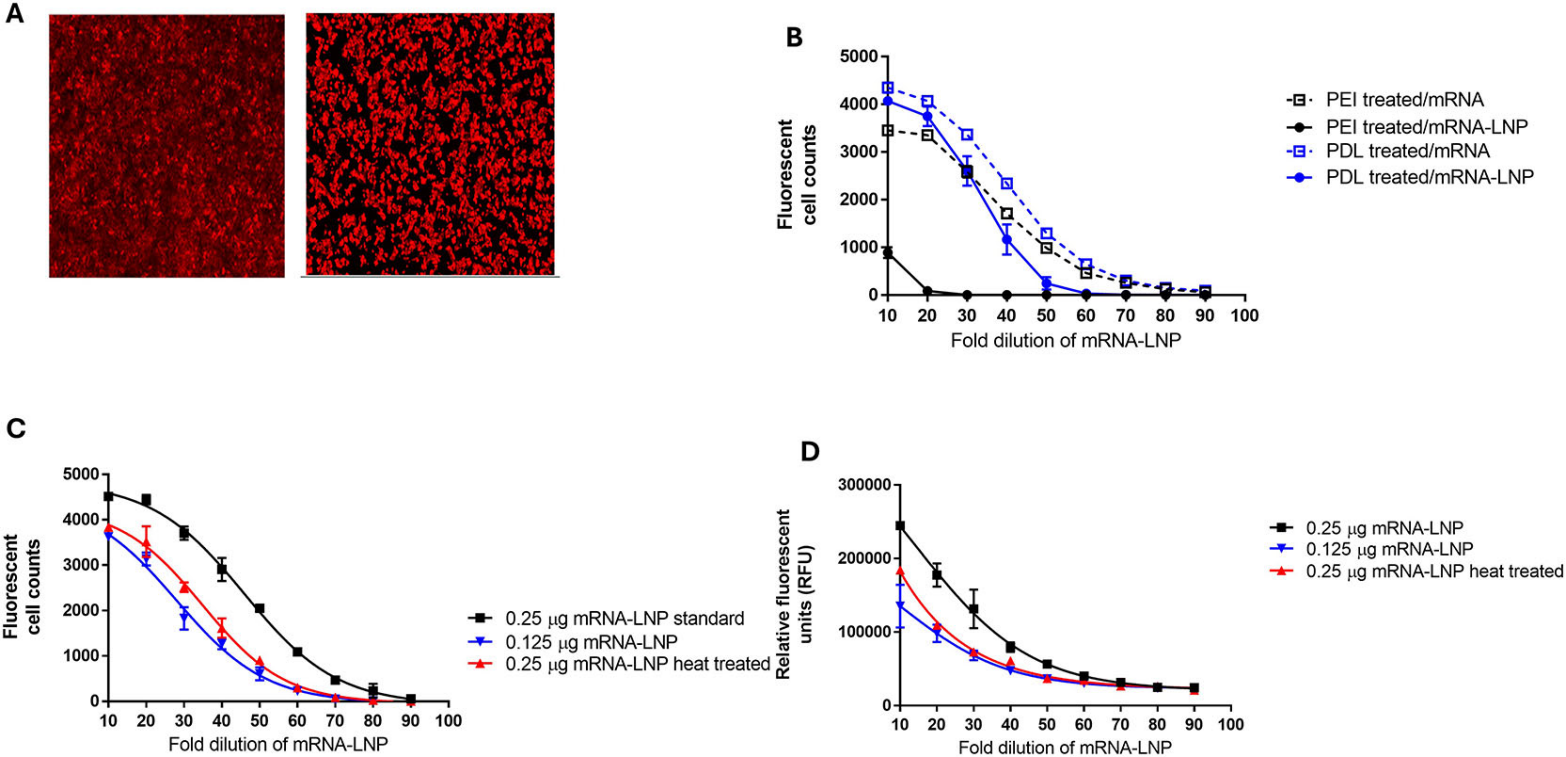
FFA and Direct ELISA development methods. A) FFA cell images of HEPG2 and HEK293 cells after mRNA-LNP exposure and measurement of protein expression, B) poly-D-lysine (PDL) and polyethylenimine (PEI) media additives effect on FFA protein measurement in HEK293 cells after mRNA and mRNA-LNP exposure, C) representative data from the FFA method testing mRNA-LNP at two different concentrations (0.25 μg and 0.125 μg) and after heat treatment at 75°C for 10 minutes, D) representative data from the direct ELISA method testing mRNA-LNP at two different concentrations (0.25 μg and 0.125 μg) and after heat treatment at 75°C for 10 minutes.

During the development of FFA and direct ELISA methods, several parameters were evaluated, including the cell line (HEK293 and HEPG2), cell concentration used for plating, and the incubation time after transfection with mRNA-LNP. Both cell lines demonstrated significant protein production after transfection with mRNA-LNPs ( Figure 3A). As HEK293 cells are semi-adherent, additional media additives, poly-D-lysine (PDL) and polyethylenimine (PEI), were evaluated to prevent loss of the cells during washing. ^15^ Both media additives prevented HEK 293 cell loss during washing, but PEI was found to interfere with mRNA-LNP transfection ( Figure 3B). Based on this work, conditions were selected for use in the FFA method, consisting of plating HEK293 cells in media containing PDL. The FFA method was sensitive to different concentrations of mRNA-LNP and stability, as shown by testing mRNA-LNP, which was subjected to heat stress at 75°C for 10 min (representative data in Figure 3C).

For the development of the direct ELISA method, cell plating and mRNA-LNP concentrations selected for the FFA method described above were applied. The amount of protein present in the direct ELISA was determined based on the total fluorescent signal for each well using an ELISA plate reader instead of counting the total number of fluorescent cells per well as done in the FFA. The direct ELISA method was sensitive to different concentrations of mRNA-LNP and stability, as shown by testing mRNA-LNP, which was subjected to heat stress at 75°C for 10 min (representative data in Figure 3D).

### Western blot

A WB method was developed for the detection of the type SARS CoV-2 spike protein generated after transfection with mRNA-LNPs. Proteins were generated in the cell culture and extracted as described above for sandwich ELISA. The test samples were then tested at different concentrations and compared to the assigned mRNA-LNP standard. The S-antigen was measured at the expected molecular weight of approximately 180 kDa (data not shown), and band intensity was used for semi-quantitation. The developed WB method was shown to be sensitive to different concentrations of mRNA-LNP as well as stability, as shown by testing mRNA-LNP subjected to heat stress at 75°C for 10 min.

### Sandwich ELISA

**Figure 4.**
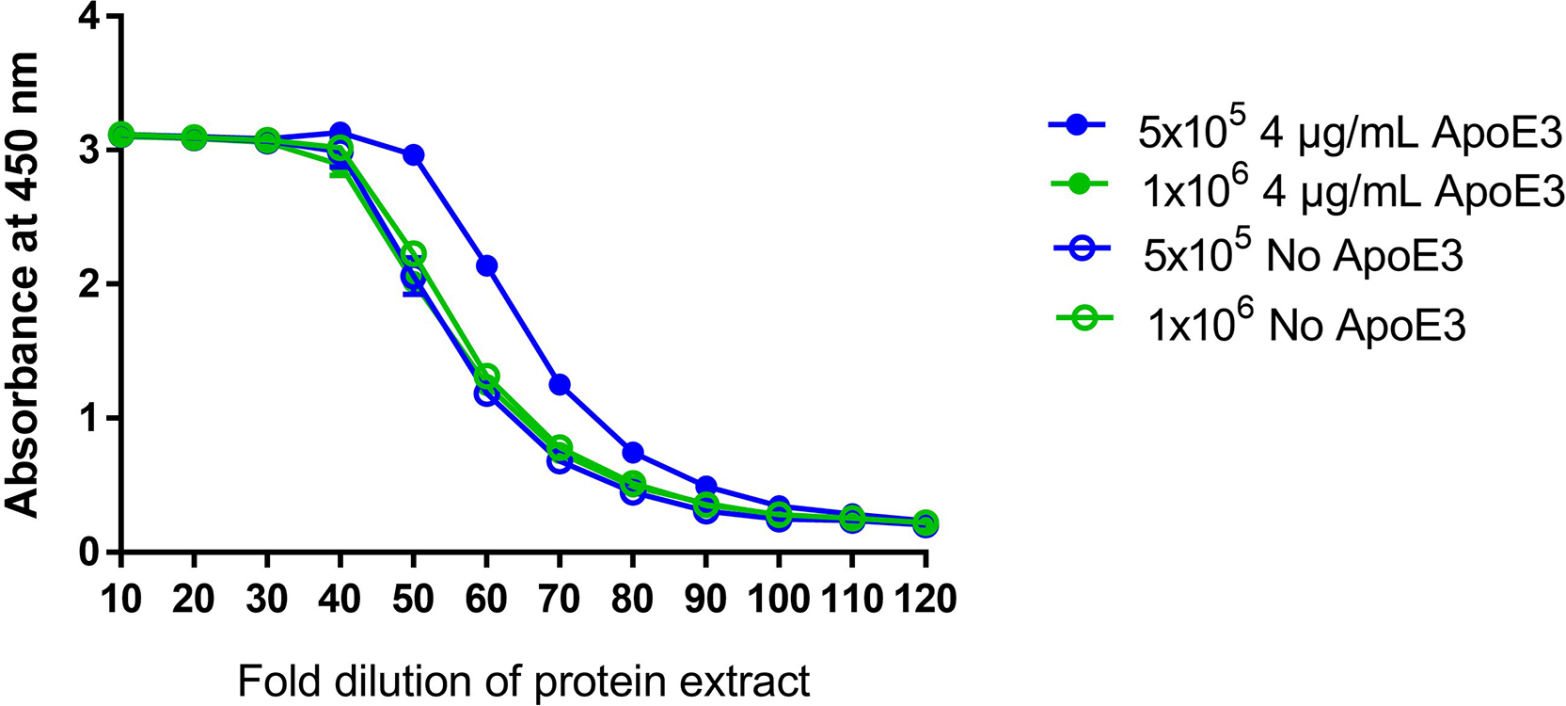
Effect of ApoE3 on detectable protein by Sandwich ELISA. Effect of addition of 4 μg of ApoE3 on the amount of detectable protein generated in two different cell concentrations (1 x10 ^6^ and 5 x10 ^5^ cells/mL) by sandwich ELISA.

To develop the sandwich ELISA, cell incubation time after transfection with mRNA-LNP, cell culture plate size, and the addition of ApoE3 were evaluated. Initially, 24-well plates were used to evaluate different assay parameters, but the final method was transitioned to 12-well plates to align with the USP guidelines for mRNA-LNP cell-based potency methods. The amount of detectable protein after transfection with mRNA-LNP was evaluated for 1 and 2 days, which meets both logistical considerations for assay conduct and is consistent with the existing mRNA-LNP potency methods. Consistent with previously published methods, we evaluated 18- and 24-hour overnight incubation times after transfection with mRNA-LNPs and observed no difference in the amount of protein generated (data not shown). ^16^^,^^17^ ApoE3 was evaluated at different concentrations (1, 2, and 4 μg) during incubation with mRNA-LNP, based on both the manufacturer’s recommendations and the USP guidelines (data not shown). ^8^^,^^18^ After overnight incubation, the protein was extracted for testing by sandwich ELISA. The addition of ApoE3 to the cell culture media increased the amount of protein detected in cells plated at a lower concentration of 5 × 10 ^5^ cells/well, but had no effect on the amount of detectable protein in cells plated at a higher concentration of 1 × 10 ^6^ cells/well ( Figure 4). As ApoE3 was shown to have minimal benefit in this test, it was not used in the final assay format.

**Figure 5.**
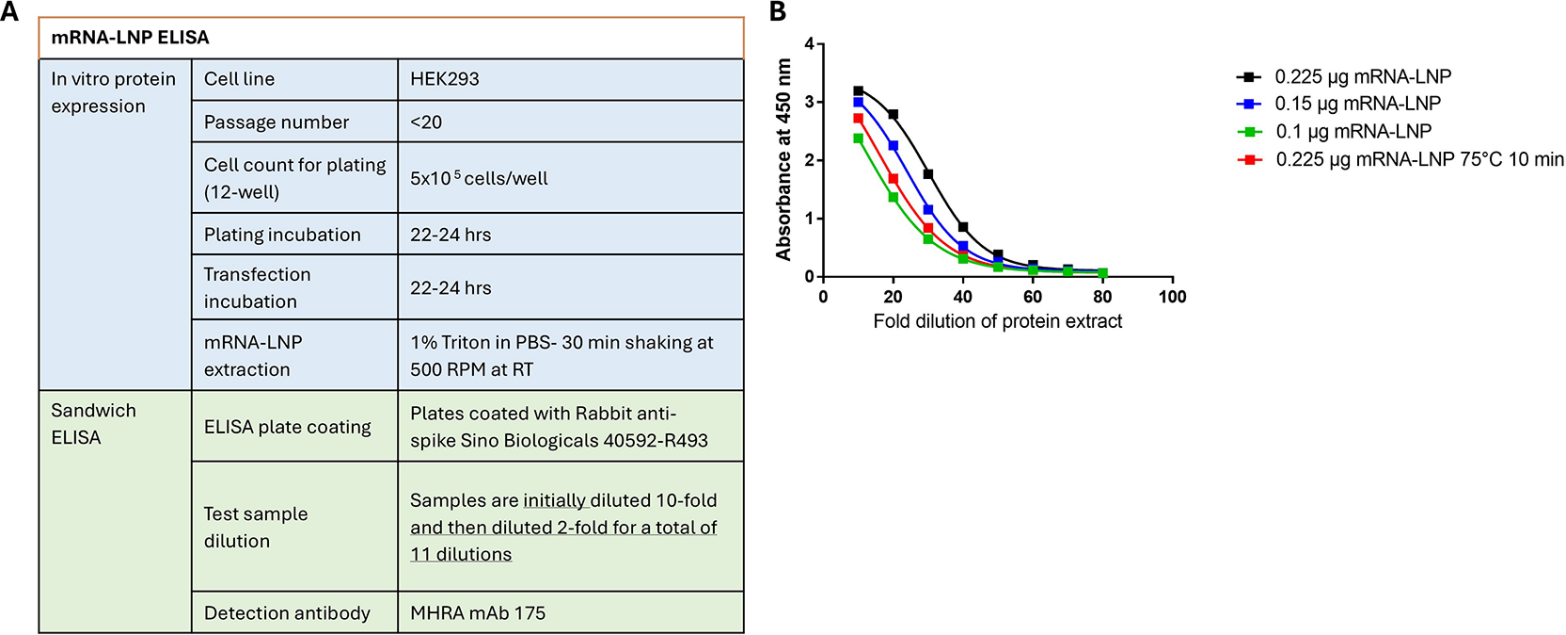
Sandwich ELISA parameters. Final assay parameters tested for qualification (A) and Representative sandwich ELISA curves of a concentration range and heat stressed for SARS-CoV-2 WT and Omicron mRNA-LNP (B).

Based on the selected sandwich ELISA conditions ( Figure 5A) representative sandwich ELISA curves are shown in Figure 5B. The sandwich ELISA demonstrated sensitivity to different mRNA-LNP concentrations, as well as stability, as shown by testing samples subjected to heat stress at 75°C for 10 min.

### Qualification

**Figure 6.**
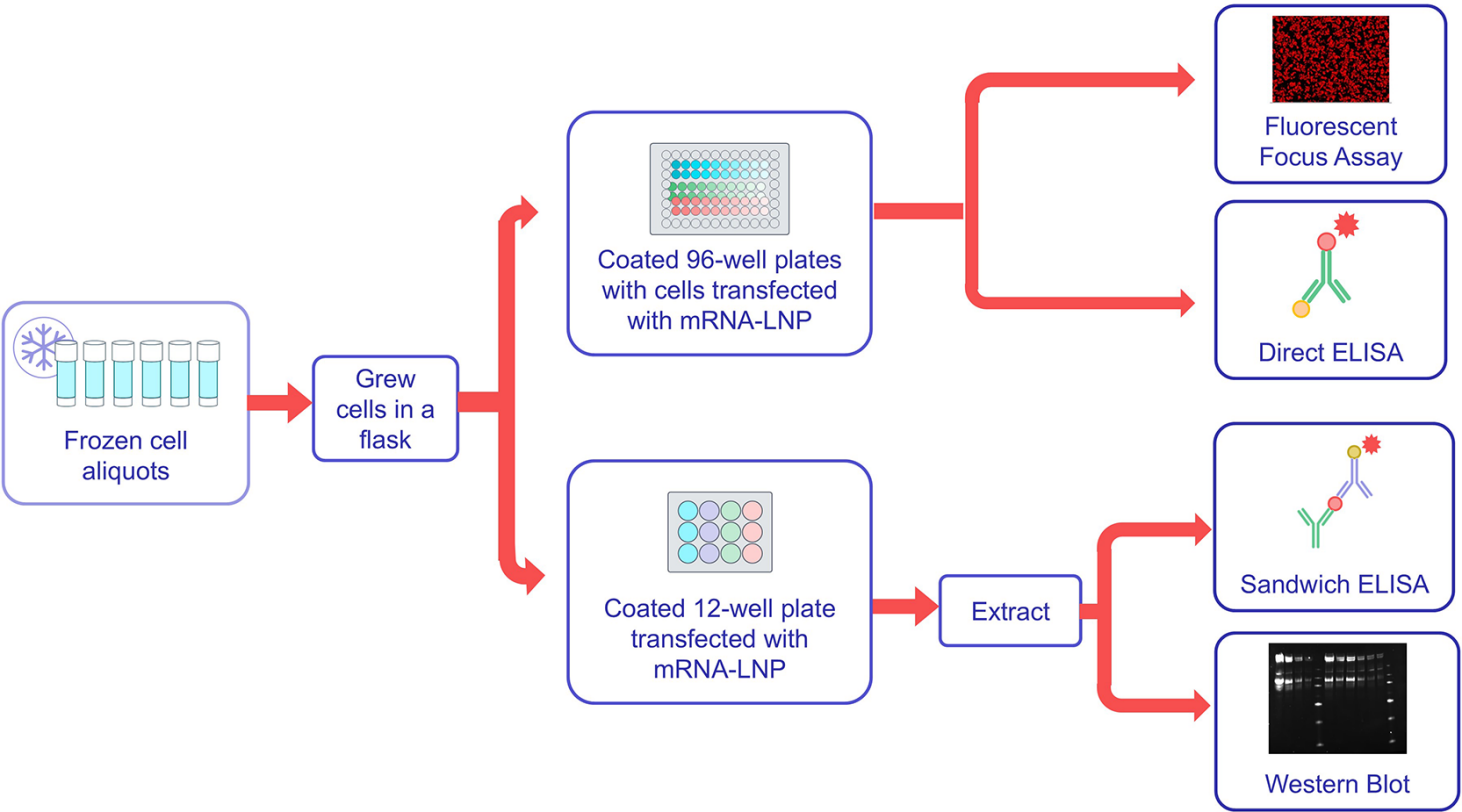
Qualification testing flow chart. As shown testing by FFA, sandwich ELISA, direct ELISA and western blot were conducted for two operators using the same panel of test samples.

Based on the sensitivity to low concentrations of mRNA-LNP, the sandwich ELISA was selected for qualification to better understand the method performance. A flow diagram of qualification testing is shown in Figure 6, and representative data from the qualification are shown in Figure 6. Qualification testing was conducted by two analysts using the wild-type SARS CoV-2 mRNA-LNP variant as the test sample across three different starting concentrations. Testing was conducted in triplicate on each testing day by each analyst, with three testing days conducted per analyst. This resulted in three cell culture plates per analyst per testing day, and each cell culture plate resulted in three ELISA plates, for a total of nine ELISA plates per operator per test day.

**Figure 7.**
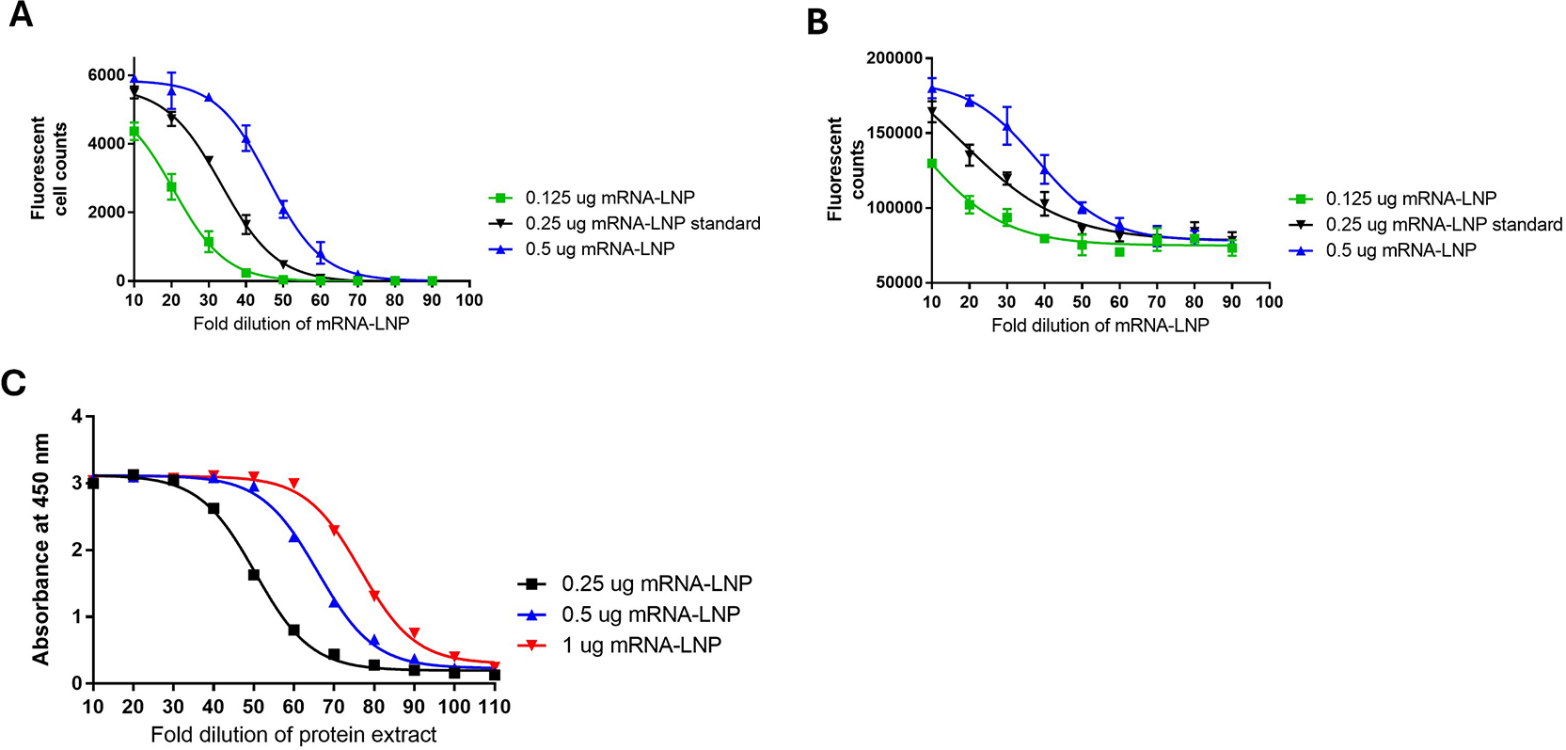
Representative potency data from SARS-CoV-2 WT mRNA-LNP qualification testing. Test samples of SARS-CoV-2 WT mRNA-LNP at 0.25 μg, 0.5 μg and 1 μg were compared to a 0.5 μg standard in all test methods. A) FFA, B) Direct ELISA, C) Sandwich ELISA.

**Figure 8.**
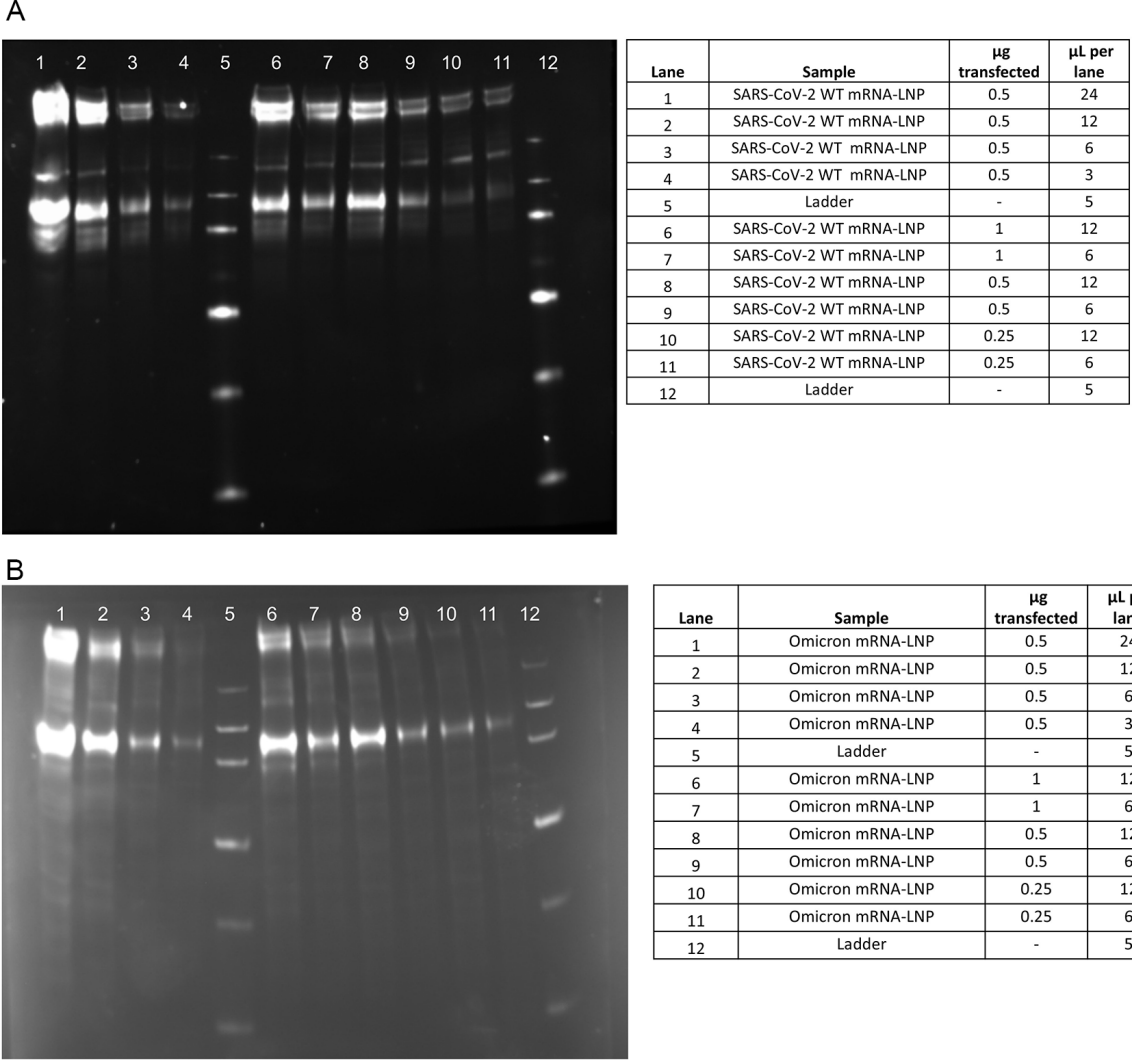
Qualification western blot. A) SARS-CoV-2 WT mRNA-LNP B) Omicron mRNA-LNP extracts with a 0.5 μg 2-fold standard dilution curve and a high and low sample load for each of the transfection amounts of 1 μg, 0.5 μg and 0.25 μg.

**Figure 9.**
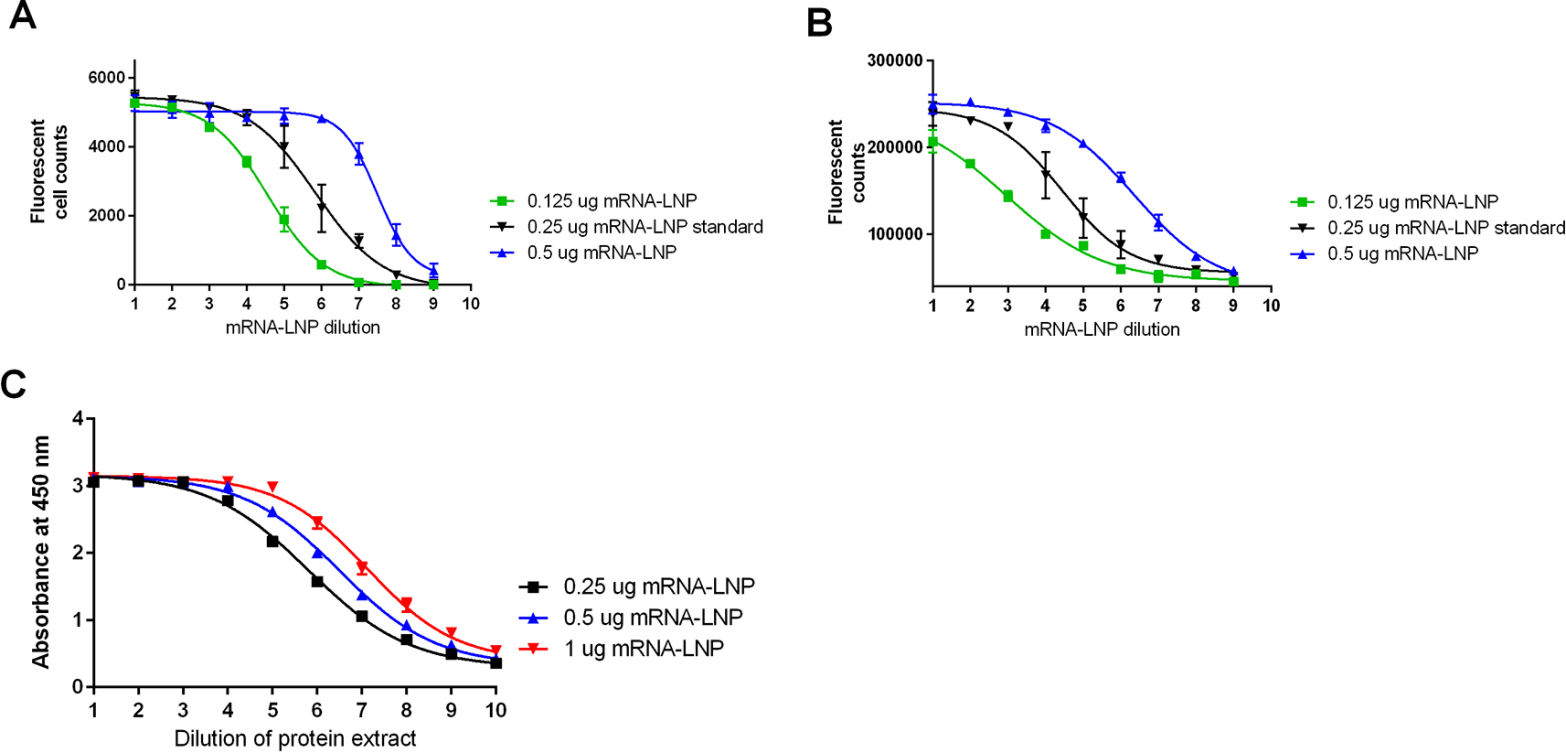
Representative potency data from Omicron mRNA-LNP qualification testing Test samples at starting concentrations of 0.25 μg, 0.5 μg and 1 μg were compared to a 0.5 μg standard. Representative data from all test methods A) FFA, B) Direct ELISA, C) Sandwich ELISA.

Testing was also conducted to evaluate the performance of the method and to better understand how this method relates to existing alternate potency methods: FFA, direct ELISA, and western blotting ( Figures 7 and 8). Additional pilot testing was conducted using the Omicron mRNA-LNP variant to demonstrate the flexibility of the developed potency methods ( Figure 9).

### Statistical analysis of sandwich ELISA qualification data

**Figure 10.**
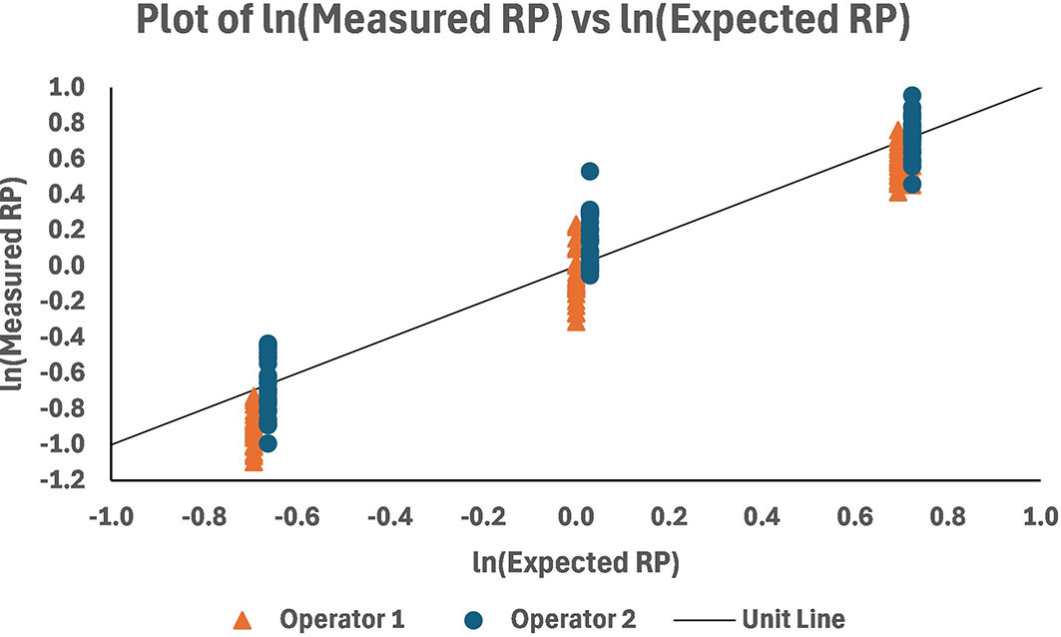
ln (Measured RP) versus ln (Expected RP) across levels; with the unit line.

A plot of the RP results (the average across plates) for the two operators over the qualification sample levels is shown in Figure 10. Graphically, there appears to be a good match with the unit line [the line passing through (0,0) with a slope equal to 1.0]. The spread of measurements is uniform at all levels within operators, while there appears to be higher results for Operator 2 versus Operator 1 across levels.

### Relative accuracy

**Figure 11.**
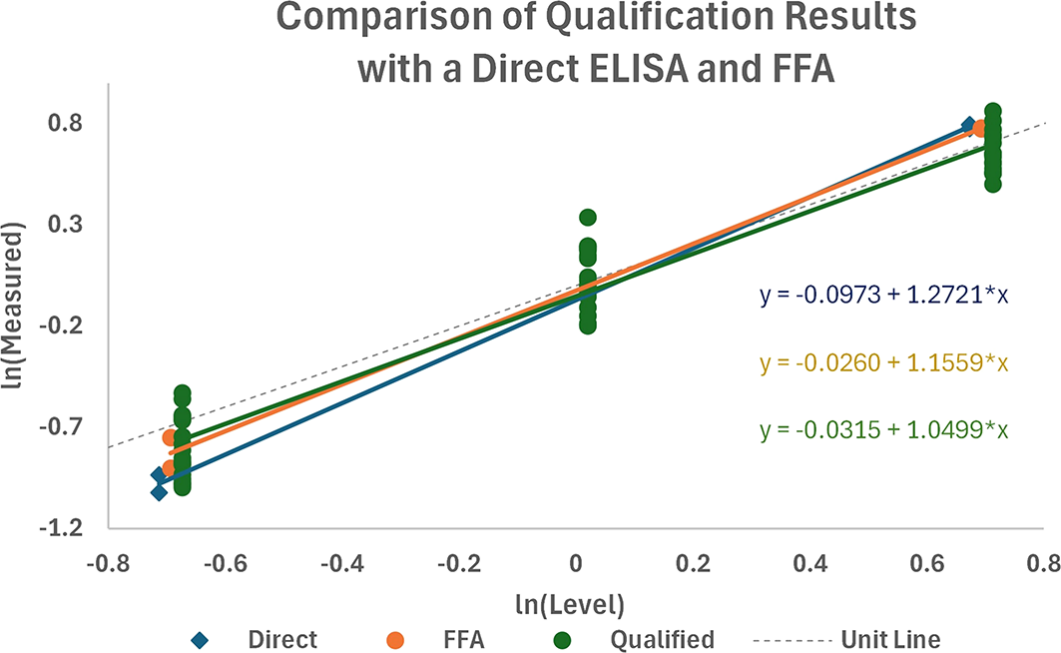
Relationships of ln (Observed RP) versus ln (Expected RP) for data obtained in the qualified ELISA, a Direct ELISA, and FFA.

MEM analysis was performed, yielding ln transformed estimates of the measured RP and their standard errors of estimate (StdErr). These were used together to determine the percent relative bias (%RB) and 95% confidence limits at each level. The estimated intercept of the model and its standard error are used to calculate the overall %RB and its limits. The limits were calculated on a ln scale and retransformed to the %RB scale. The results of the calculations for the %RB are presented in Table 3. The range in %RB is -10.2% to 5.1%, with high negative %RB at the 0.5 level due to lower-than-expected RP for Operator 1 (see Figure 11). The overall %RB across levels was -3.0% with 95% confidence interval (-19.8%, 17.2%).

**Table 3.** Percent relative bias of the assay.

	ln transformed			Relative bias		
Expected level	Expected level	Measured level	Std Err	RB	LCL	UCL
0.5	-0.6931	-0.8004	0.0965	-10.2%	-25.8%	8.7%
1	0.0000	0.0498	0.0965	5.1%	-13.2%	27.2%
2	0.6931	0.6577	0.0965	-3.5%	-20.3%	16.8%
All	0.0000	-0.0310	0.0958	-3.0%	-19.8%	17.2%

### Repeatability, intermediate precision, and reportable result variability

MEM analysis also yielded variance component estimates that were used to establish the repeatability and intermediate precision (IP) of the method. The calculations were performed at each level and overall ( Table 4). Repeatability and intermediate precision (%GCV) at individual levels ranged from 11% to 16% and 15% to 23%, respectively, with high IP at the 0.5 level due to lower-than-expected RP for operator 1 ( Figure 11). The overall repeatability was 11% GCV and intermediate precision was 21%. Fifty percent of the overall IP is due to interoperator variability.

The %GCV for a reportable result for replication strategies with i-operators, j-days, k-cell culture preparations per operator and day, and n-replicates was calculated using the overall estimates of the variance components. The results are presented in Table 5. The greatest reduction in variability can be observed by using multiple operators (e.g., the predicted %GCV of the reportable result decreases from 21% to 14% when the procedure is performed using 2-operators), which is consistent with the observation that operator accounts for nearly 50% of the overall variability.

**Table 4.** Predicted %GCV for a reportable result.

			#Replicates			
#Operators	#Days	#Preps	n = 1	n = 2	n = 3	n = 6
i = 1	j = 1	k = 1	21%	19%	18%	17%
i = 1	j = 1	k = 2	18%	17%	16%	16%
i = 1	j = 2	k = 1	18%	17%	16%	16%
i = 1	j = 2	k = 2	16%	15%	15%	15%
i = 2	j = 1	k = 1	14%	13%	12%	12%
i = 2	j = 1	k = 2	12%	11%	11%	11%
i = 2	j = 2	k = 1	12%	11%	11%	11%
i = 2	j = 2	k = 2	11%	11%	11%	10%

**Table 5.** Repeatability and intermediate precision of the assay.

	Variance components (natural log scale)						%GCV	
Level	Operator	Day	Day*Operator	Cell culture (Day*Operator)	Residual	Total	Repeatability	IP
0.5	0.0245	0	0.0012	0.0041	0.0132	0.0431	14%	23%
1	0.0223	0	0.0000	0.0076	0.0137	0.0436	16%	23%
2	0.0078	0	0.0000	0.0026	0.0084	0.0189	11%	15%
Overall	0.0176	0	0.0000	0.0059	0.0113	0.0348	11%	21%
(%Total)	(50%)	(0%)	(0%)	(17%)	(33%)			

### Linearity

**Figure 12.**
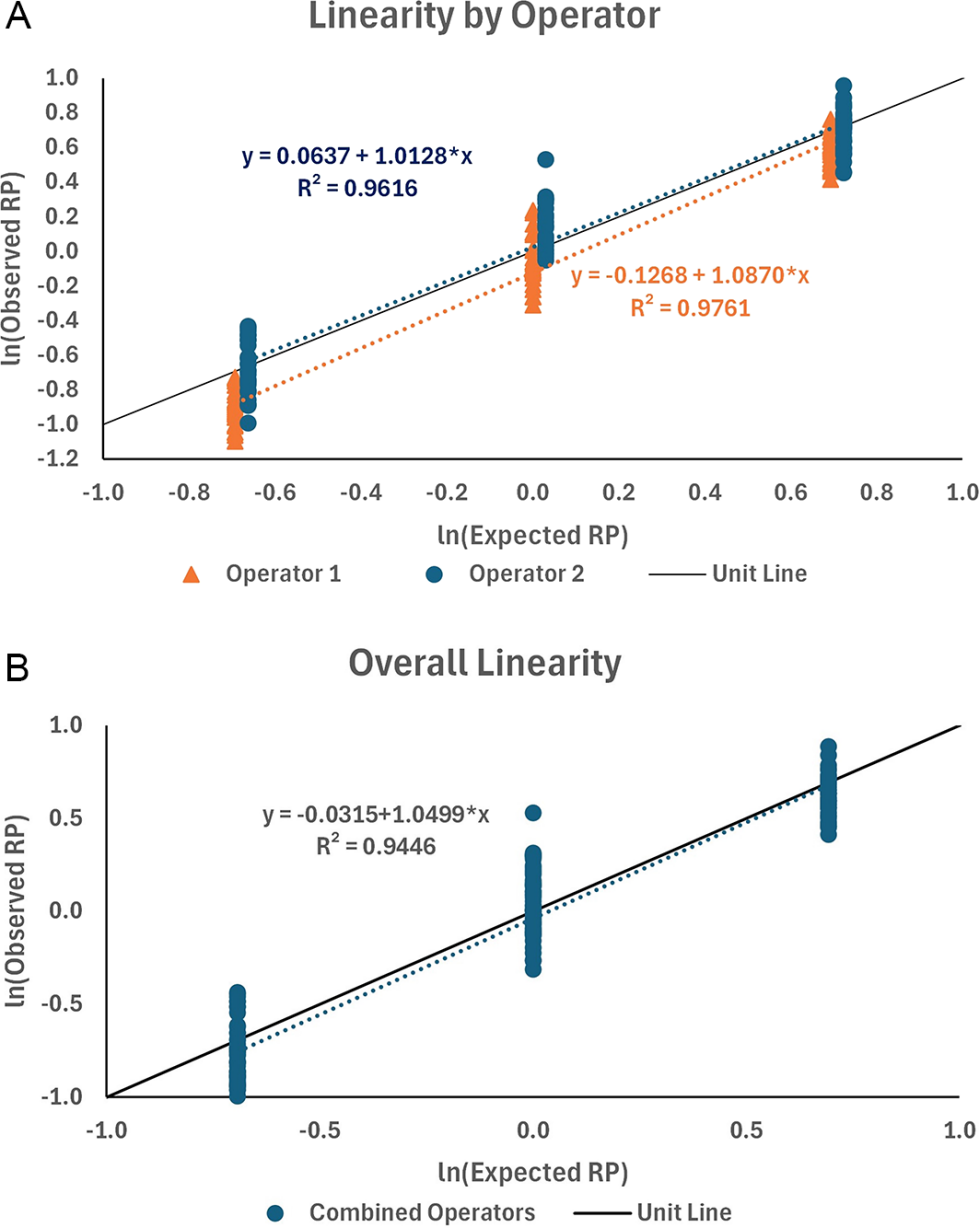
Linear fits to ln (Observed RP) versus ln (Expected RP) by operator and overall.

The data from qualification testing were fit using linear regression, first by operator and then for the combined data. Plots of the data and their linear fits along the unit line are shown in Figure 12. The linear regression for the two operators showed a good fit (R ^2^ = 0.976 and 0.962 for operators 1 and 2, respectively) with slopes close to 1.0 (b = 1.0870 and 1.0128 for operators 1 and 2, respectively). The data were combined between the two operators, resulting in an acceptable fit of the linear regression (R ^2^ = 0.945) and slope (b = 1.0499). The slope was used to determine the proportional bias (PB) in the results with span f = 2.0-fold. That is, the fold difference between results may be biased from the true fold 2-fold difference by as much as 3.5%. The percentage proportional bias for the other fold differences (f ) is presented in Table 6.

**Table 6.** %PB for given fold differences (f).

f	%PB
1.0	0.0%
1.2	0.9%
1.4	1.7%
1.6	2.4%
1.8	3.0%
2.0	3.5%

### Comparisons to a direct binding ELISA and FFA

The sandwich ELISA qualification data were combined with the data from direct ELISA and FFA, as shown in Figure 11. A preliminary assessment of the differences in profiles showed no significant differences in the slopes of the regressions (P = 0.100) or among the level of responses (P = 0.667), suggesting that there was no significant difference between the sandwich ELISA and the direct ELISA and FFA methods with this test sample panel.

## Discussion

Existing mRNA potency methods, such as flow cytometry, FFA, or WB, are challenging in an LMIC vaccine manufacturing setting, and alternative potency methods are needed. Flow cytometry methods require complex equipment and are challenging to validate in manufacturing settings. FFA methods, either manual cell counting or as described in this manuscript, require specialized equipment with counting software (Spectramax i3X plate reader with the Minimax attachment (Molecular Devices)). Finally, WB methods are semi-quantitative and may have limited sensitivity compared with ELISA potency methods. Here, we describe the development of both direct and sandwich ELISA methods that allow for quantification of the protein generated after in vitro transfection in cell culture. Both methods were shown to be stable, and the developed sandwich ELISA method was able to better understand assay performance characteristics.

HEK293 cells were selected for use in all developed methods because of the increased protein expression observed after a 24-hour transfection incubation period. As both FFA and direct ELISA methods require cell culture plate washing, HEK293 cells, which are semi-adherent, were lost during washing. To address this cell loss, a medium additive to improve cell adherence was identified. Both PEI and PDL were evaluated; however, PEI was shown to interfere with mRNA-LNP protein generation after transfection. This interference may be due to PEI acting as a detergent and disrupting the mRNA-LNP transfection. Additional studies are required to investigate this observed interference.

Potency methods for mRNA-LNP methods require measurement of the protein generated by mRNA-LNP candidates. To address this, a two-part assay format was developed, including in vitro cell culture for transfection and protein expression of the mRNA-LNP, followed by protein measurement from extracted samples in the sandwich ELISA. All the methods evaluated require in vitro cell culture for protein generation, which adds to the assay complexity and duration. An additional limitation is the requirement for antigen-specific antibodies for detection. Direct ELISA requires one target-specific antibody, whereas sandwich ELISA requires two. However, the sandwich ELISA appeared to be more sensitive to lower concentrations of mRNA-LNP. As RNA vaccine development advances, lower amounts of RNA may be required to achieve a clinically relevant outcome. This may be particularly important as the field moves from mRNA vaccines with doses in the 50 μg range to self-amplifying RNA vaccines, which can be effective at much lower doses in the 10 μg range. Sandwich ELISA may allow better detection of lower RNA concentrations. The development of antigen-specific antibodies requires time and investment, which are challenging when attempting to respond to a pandemic threat. However, antigen-specific antibodies are still critical for confirming that an appropriate protein is generated to ensure the intended clinical outcome. A limitation of this study is a lack of a mock transfection control in the experimental design. Future adaptations of these methods for application to vaccine candidates will require additional testing for potential interference or non-specific binding using the antibody specific for the target of interest.

The developed ELISA techniques provide quantitative methods to measure protein production for mRNA-LNP vaccine candidates in the lab. As *in vitro* cell culture transfection is not an accurate mimic of *in vivo* transfection efficiency the methods were designed as relative potency which compares protein production in test samples to a known reference standard, similar to other relative potency methods, rather than calculated protein produced on a per cell basis. Both ELISA techniques developed do not provide a quantitative measurement of protein expression per cell (i.e., micrograms of antigen produced per number of transfected cells). The FFA can offer some level of quantification of protein expression per cell as described by Li, H. H. et al. although the relevance of this *in vitro* measure to *in vivo* transfection efficiency needs to be evaluated. ^16^ In addition, a single antibody is used for detection in the developed ELISAs and this does not confirm fully intact protein complexes. Additional antibodies could be evaluated in future work to ensure sensitivity to complete S-antigen protein complexes or relevant neutralizing epitopes. As this work was focused on development of a platform potency assay utilizing SARS CoV-2 as model vaccine additional antibodies were not explored as part of this project. Additional work is ongoing to conduct full characterization including epitope mapping for the selected monoclonal antibody 175 which will be published along with partners.

Qualification was performed to better understand the performance of the two-part method. The Sandwich ELISA demonstrated overall intermediate precision of 21%, with number of operators having the largest impact. This suggests that future applications of this method should be conducted by multiple operators to improve intermediate precision.

The sandwich ELISA was shown to be comparable to FFA, WB, and direct ELISA methods for testing selected mRNA-LNP samples. In addition, the adaptability of the sandwich ELISA was demonstrated by testing both wild type SARS CoV-2 and Omicron mRNA-LNP targets. Future application of the sandwich ELISA to mRNA-LNP targets other than SARS CoV-2 would require replacing antibodies with reagents specific to the target of interest. Target ranges were identified for several of the method parameters in an attempt to standardize the method and limit the amount of optimization required for application to other vaccine targets. As SARS CoV-2 was selected as a model vaccine candidate for method development only heat testing was conducted at this time with the intention for manufacturers to adapt the developed methods to other mRNA-LNP targets in development. Additional testing would be required according to ICH standards to demonstrate full sensitivity to stability changes of the method with the true vaccine candidate.

A quantitative potency assay was developed to accurately measure the mRNA-LNP spike protein production in HEK293 cells. The sandwich ELISA was shown to be stable and qualified to better define assay performance parameters. Comparability with other test methods was conducted, and it was confirmed that the performance of the developed sandwich ELISA was comparable with WB, FFA, and direct ELISA methods. The adaptability of the sandwich ELISA to other mRNA-LNP candidates was demonstrated by testing a commercial mRNA-LNP vaccine targeting the Omicron variant.

## Data Availability

Repository: Qualification of quantitative stability indicating potency assay for mRNA-LNP vaccine candidates. https://doi.org/10.17605/OSF.IO/MV4RK This project contains the following underlying data:
• Data file 1. Statistical analysis of Sandwich ELISA results • Data file 2. Representative antibody screening data by ELISA • Data file 3. Effect of cell line and extraction buffer on protein concentration • Data file 4. FFA and Direct ELISA development methods • Data file 5. Effect of ApoE3 on detectable protein by Sandwich ELISA • Data file 6. Sandwich ELISA parameters • Data file 7. Representative potency data from SARS-CoV-2 WT mRNA-LNP qualification testing • Data file 8. Representative potency data of Omicron mRNA-LNP qualification testing Data are available under the terms of the Creative Commons Zero “No rights reserved’ data waiver (CC0 1.0 Public domain dedication).
